# Role of Neural Stem Cell Activity in Postweaning Development of the Sexually Dimorphic Nucleus of the Preoptic Area in Rats

**DOI:** 10.1371/journal.pone.0054927

**Published:** 2013-01-30

**Authors:** Zhen He, Sherry A. Ferguson, Li Cui, L. John Greenfield, Merle G. Paule

**Affiliations:** 1 Division of Neurotoxicology, National Center for Toxicological Research, Food and Drug Administration, Jefferson, Arkansas, United States of America; 2 Department of Neurology, University of Arkansas for Medical Sciences, Little Rock, Arkansas, United States of America; Hosptial Infantil Universitario Niño Jesús, CIBEROBN, Spain

## Abstract

The sexually dimorphic nucleus of the preoptic area (SDN-POA) has received increased attention due to its apparent sensitivity to estrogen-like compounds found in food and food containers. The mechanisms that regulate SDN-POA volume remain unclear as is the extent of postweaning development of the SDN-POA. Here we demonstrate that the female Sprague-Dawley SDN-POA volume increased from weaning to adulthood, although this increase was not statistically significant as it was in males. The number of cells positive for Ki67, a marker of cell proliferation, in both the SDN-POA and the hypothalamus was significantly higher at weaning than at adulthood in male rats. In contrast, the number of Ki67-positive cells was significantly higher in the hypothalamus but not in the SDN-POA (p>0.05) at weaning than at adulthood in female rats. A subset of the Ki67-positive cells in the SDN-POA displayed the morphology of dividing cells. Nestin-immunoreactivity delineated a potential macroscopic neural stem cell niche in the rostral end of the 3rd ventricle. In conclusion, stem cells may partially account for the sexually dimorphic postweaning development of the SDN-POA.

## Introduction

The sexually dimorphic nucleus, involved in the regulation of sexual behavior, has been defined in human, nonhuman primates, and other species, including the sexually dimorphic nucleus of the preoptic area (SDN-POA) in rats [1]. Volume of the adult male rat SDN-POA is typically 3–8 times that of the female [1], [2]. This marked sex difference in volume is due principally to an increase in the total area of higher cell and neuronal density in adult males [1]. Similarly, the sexually dimorphic nucleus of adult men contains a higher total cell number relative to adult women [3].

Initial measurements of the SDN-POA were conducted using the Nissl method which delineated SDN-POA boundaries by staining the negatively charged RNA blue with thionin or cresyl violet. The method is still acceptable and in use [4]. Nonetheless, there is increasing evidence that the calbindin-D28K (CB28) immunoreactivity-delineated nucleus-like structure located in the preoptic area can be used to determine SDN-POA volume, partially because CB28 immunoreactivity provides a clearer boundary which is more easily distinguishable from the surrounding CB28 immunoreactivity-negative structures [5]. Further, a similarly sexually dimorphic SDN-POA area in mice cannot be delineated by Nissl staining [6], [7], but is distinguishable using CB28 immunoreactivity [7]–[10]. Originally, the CB28-delineated SDN-POA was considered a subdivision of the SDN-POA determined using the Nissl method [11]. However, the CB28-delineated area is now often used as a proxy for the SDN-POA [12].

We recently described a significant increase in SDN-POA volume in postnatal day (PND) 21 male rats that were developmentally treated with low doses (2.5 or 25.0 µg/kg/day) of bisphenol A (BPA) [5], a potential endocrine disrupter. BPA treatment did not alter SDN-POA volume of PND 21 females; however, treatment with ethinyl estradiol (EE_2_) (10 µg/kg/day) enlarged SDN-POA volume in both sexes at PND 21, indicating the sensitivity of both sexes to this reference estrogen. Nonetheless, the mechanisms by which BPA and EE_2_ treatment increased the size of the SDN-POA remain unclear. In fact, many of the originally proposed mechanisms for SDN-POA volume differences have not been substantiated by recent studies. For example, there do not appear to be sex differences in SDN-POA prenatal neurogenesis, neuronal migration, and apoptosis [12], [13]. Thus, postnatal processes may be of particular importance in defining the ultimate SDN-POA area. Given the plasticity of adult SDN-POA volume [14] and the continued development of the SDN-POA postweaning [15], those postnatal processes may likely extend beyond the postweaning period.

The present study evaluated postweaning development of the SDN-POA in male and female weanling (PND 21) and adult rats. The SDN-POA territory was defined using CB28 immunoreactivity and/or a cellular nucleus-staining method with DAPI (4′,6-diamidino-2-phenylindole) which defines the SDN-POA as a congested nuclear mass in the hypothalamus as we recently reported [5]. Simultaneously, stem cell activity in the SDN-POA and surrounding area was assessed using four well-recognized stem cell markers: nestin (a type VI intermediate filament protein as a marker of proliferating and migrating cells) [16, http://en.wikipedia.org/wiki/Nestin_(protein)]; Ki67 (an indicator of proliferative/mitotic activity) [16], [17]; SOX2 (a transcription factor essential for maintaining self-renewal or pluripotency) [17]; and CD133 (a glycoprotein expressed in several types of stem cells including those for neurons and glia) [18].

## Materials and Methods

### Animals

All animal procedures were approved by the National Center for Toxicological Research (NCTR) Institutional Animal Care and Use Committee. Male and female Sprague-Dawley rats were obtained from the NCTR breeding colony. At weaning (PND 21) or adulthood (PND 110), each rat was anesthetized using pentobarbital (i.p.) and sacrificed by intra-arterial perfusion of 100 ml of saline followed by 100 ml of 4% buffered paraformaldehyde. The brain was sectioned coronally into 30 µm thick slices and collected in 3 series, each having a 90 µm interval along the brain longitudinal axis between any two adjacent slices. A total of 24 brains were evaluated (n = 6/sex/age).

### Delineation of the SDN-POA

A triple fluorescent immunoreactivity labeling method [5] was utilized with slight modification [19], [20] to define the SDN-POA using one of three series of brain slices. CB28 (rabbit anti-CB28 antibody, Sigma Chemical Company, St. Louis, MO) and NeuN (neuronal nuclear antigen, mouse anti-NeuN, Chemicon/Millipore, Billerica, MA) were used as the primary antibodies. Secondary antibodies were Alexa 488-labeled goat anti-rabbit and Alexa 594-labeled goat anti-mouse (Molecular Probes/Invitrogen Carlsbad, CA). DAPI was utilized for visualizing cell nuclei. Digital images were acquired using an Olympus digital camera (DP72). The CB28 immunoreactive-labeled area was determined using NIH Image J software and the volume was calculated as previously described [5], [21].

### Determination of Neural Stem Cell Activity in the SDN-POA and Hypothalamus

A triple fluorescent immunoreactivity labeling method was utilized to define stem cell activity in the SDN-POA and hypothalamus as described earlier with slight modification [22], [23]. Stem cell activity was assessed by measuring the expression of stem cell-specific proteins, proliferative cell counts, and cell division morphology when associated with the proliferative/mitotic marker. The triple labeling method included a combination of one primary antibody of mouse anti-nestin or -Ki67, one primary antibody of rabbit anti-Ki67, -SOX2, -CD133 or -CB28 and the nucleic staining with DAPI. Fluorescent goat anti-mouse and goat anti-rabbit antibodies (Molecular Probes/Invitrogen, Carlsbad, CA) served as the second antibody, respectively. Since the coronal brain slice series containing the SDN-POA and hypothalamus also included the lateral ventricles, the subventricular zone (SVZ) served as an internal positive control for defining stem cell activity/immunoreactivity. In fact, mouse antibody against nestin (1∶100, BD Pharmingen/BD Biosciences, San Jose, CA) and mouse antibody against Ki67 (1∶300, BD Pharmingen/BD Biosciences, San Jose, CA) and rabbit against CB28 (see earlier) successfully defined the antigen-specific immunoactivities. The remaining antibodies (rabbit antibodies against Ki67, SOX2, or CD133), all purchased from commercially available sources, failed to generate reliable antigen-specific immunoreactivity in the stem cell-positive region of the SVZ. Respectively, the number of the cells with Ki67 immunoreactivity within the SDN-POA and hypothalamus was counted per slice and the area of the SDN-POA and hypothalamus was subsequently measured accordingly using NIH Image J software as described previously [24], [25]. A clear-cut CB28-positive cell mass was recognizable as defining the SDN-POA over a length of 90 microns<X <270 microns (in 1∼3 sequential slices) along the brain longitudinal axis of weanling and adult, female and male rats in the present study. The Ki67-positive cell count was restricted to the SDN-POA as defined by the presence of CB28 immunoreactivity. Briefly, Ki67-immunoreactive nuclei were counted according to the optical dissector principle [26]: once focused under a 40× lens, the red fluorescent labeling was counted only when it was found in the same cell with a blue fluorescence-outlined nucleus labeled with DAPI. Similarly, the number of Ki67-positive cells in the hypothalamus (including the SDN-POA) was counted in 5 sequential slices (90 micron intervals between slices) in a rostral to caudal direction starting at Bregma −0.40 mm. The counting was conducted in a 1.475 mm^2^ area that covered the hypothalamus bilaterally. Finally, the cell number was divided by the measured area of the region of interest (for example, hypothalamic area = 1.475 mm^2^–3^rd^ ventricle area) to generate the number of Ki67-positive cells per mm^2^. The average count derived bilaterally from the SDN-POA (1–3 sequential slices/animal) or the hypothalamus (5 sequential slices/animal) was used for statistical analyses.

### Confirmation of Fluorescent Images in a 3-dimensional View

A subset of triple labeling images, CB28-NeuN-DAPI or CB28-Ki67-DAPI, were rechecked using the Stereo Investigation System from MBF Bioscience (Williston, VT) integrated with a Zeiss AxioImager M2 microscope system and CrEST CARV II confocal imager (CCD CAMERA+CONTROLLER, ROME, ITALY) by two independent investigators. Stack images were acquired at 1 or 2 µm interval along the “Z” axis/longitudinal axis of the brain to generate 3-dimensional views of the regions of interest. NIS-Element software (Nikon, Melville, NY) was then employed to reconstruct 3-dimensional images including movies or cut-views. The Stereo Investigation System from MBF Bioscience was used to reconstruct the three dimensional views for the potential stem cell niche of the 3^rd^ ventricle.

### Fluorescent Image and Statistical Analyses

**Figure 1:**
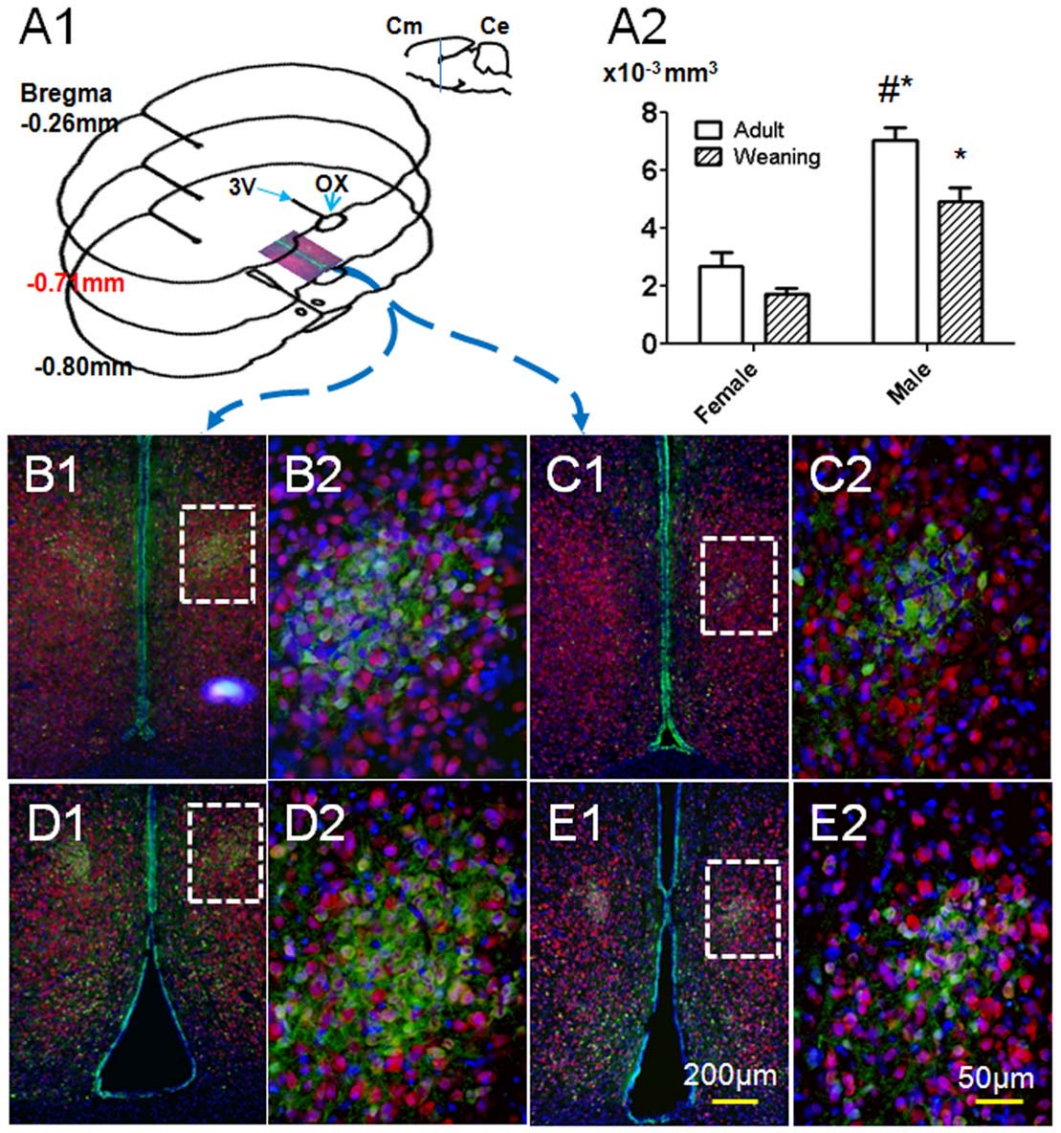
Representative images and size determination of the SDN-POA. A1: anatomical localization of the SDN-POA as delineated by CB28 immunoreactivity. Cm = cerebrum; Ce = cerebellum; 3V = 3^rd^ ventricle; OX = optic chiasm. A2: Graphic SDN-POA volumes (n = 6/sex/age, Mean±SE). #p<0.01 vs. weanling male. *p<0.001 vs. age-matched females. Panels B1, C1, D1, E1 illustrate triple-labeling images which include the bilateral preoptic area of the hypothalamus from male and female rats at postnatal day (PND) 21 and PND 110. Panels B2, C2, D2, E2 highlight the SDN-POA as was indicated in the white dotted area in panels B1, C1, D1, E1. B1&2: PND 21 male; C1&2: PND 21 female; D1&2: PND 110 male; E1&2: PND 110 female. Green fluorescence indicates calbindin D28K (CB28)-positive neurons, red fluorescence indicates NeuN-immunoreactivity, and blue (DAPI) fluorescence delineates cellular nuclei.

SDN-POA volumes were often not bilaterally symmetrical (e.g., Fig. 1C1 in which the SDN-POA is almost absent on one side) as we reported previously [5]. Accordingly, the left and right volumes of the SDN-POA were averaged for each subject as done previously [5] and this average volume was used in the statistical analyses. Average SDN-POA volumes were analyzed using a two-way ANOVA with age, sex, and the interaction as factors and posthoc tests were conducted using the Bonferroni method (GraphPad Prism 5, GraphPad Software, Inc., La Jolla, CA). Average cell counts of the Ki67-positive cells in the SDN-POA and the hypothalamus were similarly analyzed. P<0.05 was considered as significant.

## Results

As shown in Fig. 1, the SDN-POA was delineated by CB28 immunoreactivity and an associated, congested nuclear mass highlighted by DAPI. NeuN-immunoreactivity, which specifically defines neurons, did not distinguish the SDN-POA from the surrounding regions. Nevertheless, a subset of NeuN-positive cells within the CB28-delineated SDN-POA did not express CB28 and vice-versa, indicating that there are several neuronal subtypes in the SDN-POA, since CB28 immunoreactivity is also neuron-specific [27], [28]. Volumes of the female SDN-POA were 1.71±0.20×10^−3^ at PND 21 and 2.70±0.45×10^−3^ mm^3^ at PND 110 and although the adult volume was larger, this was not a statistically significant difference (Fig. 1A2). Male SDN-POA volumes were 4.91±0.48×10^−3^ and 7.03±0.45×10^−3^ mm^3^, respectively at PNDs 21 and 110 (Fig. 1A2). At each age, male volume was significantly larger than the same-age female group (p<0.0001 for sex effect). Volume was larger in adult than PND 21 males (p<0.01 for age difference). The interaction of age and sex was not significant (p>0.05).

**Figure 2:**
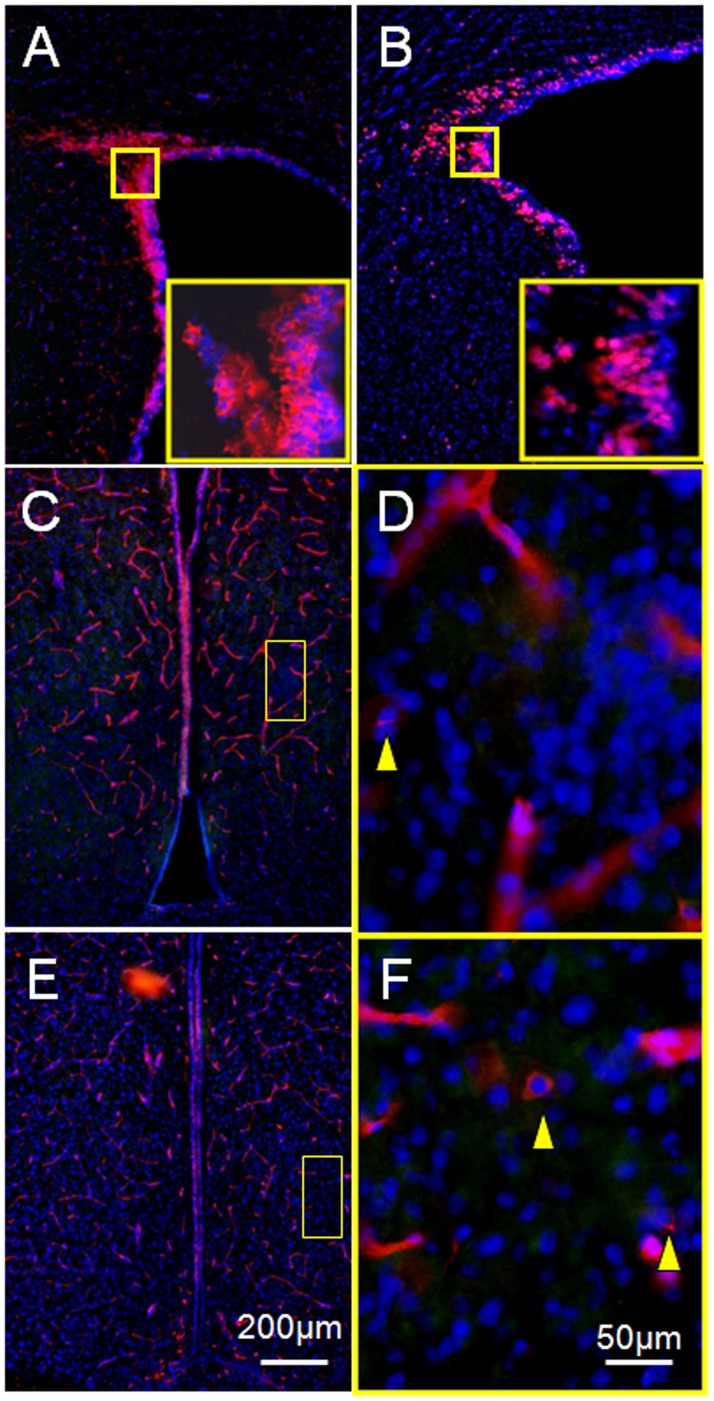
Representative images showing nestin- or Ki67-immunoreactivity in the preoptic area of the hypothalamus. Panel A displays the nestin-delineated subventricular zone (SVZ), which served as an internal positive control, while panel B shows the Ki67-labeled SVZ. Red fluorescence indicates nestin- or Ki67-immunoreactivity and blue (DAPI) fluorescence delineates cell nuclei. Insets in panels A and B are magnified images acquired at the locations indicated by the white dotted square. Panels C and E demonstrate the bilateral preoptic area of the hypothalamus labeled by nestin-immunoreactivity and DAPI from two PND 21 male animals. Images displayed under higher magnification in panels D and F were acquired at the locations indicated by the white dotted areas in Panels C and E. Arrows indicate non-vascular cells that expressed nestin. Nestin-immunoreactivity delineated the microvasculature well.

**Figure 3:**
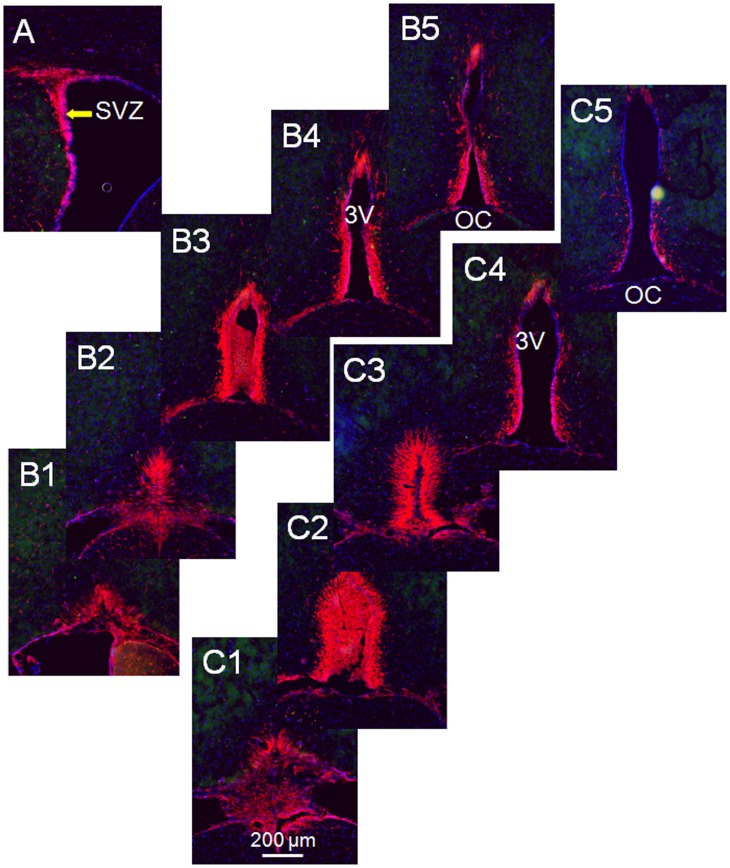
Mapping the 3rd Ventricle Stem Cell Niche, weanling vs. adult. Panel A displays the nestin-delineated subventricular zone (SVZ), which served as an internal positive control. Images in series B were acquired from a PND21 male rat and images in series C were taken from an adult male (PND110) rat, demonstrating the nestin-positive region/cells (red label) that extended into the parenchyma of the hypothalamus (∼0.1 mm or more from the ventricular wall) at the rostral end of the 3^rd^ ventricle, namely the 3^rd^ ventricle stem cell niche (3VSCN). Distance between the 2 adjacent brain slices is 90 µm. SVZ, subventricular zone; OC, optic chiazma 3V, the 3rd ventricle; blue labelings, DAPI-staining.

**Figure 4:**
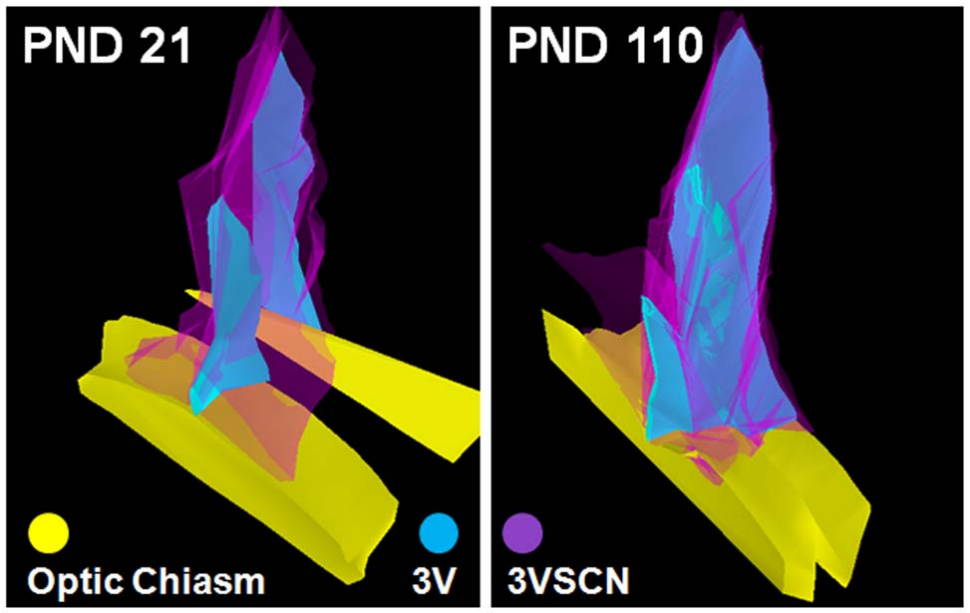
Reconstruction of 3-dimensional images of the 3VSCN. The sequential images demonstrated in Fig. 3 were employed to reconstruct 3-dimensional images of the 3VSCN using the Stereo Investigation software from MBF Bioscience. 3VSCN, the 3rd ventricular stem cell niche; PND, postnatal day; 3V, the 3rd ventricle.

Stem cell markers, nestin or Ki67, independently produced clear definition of the subventricular zone (SVZ), a prominent stem cell area in the brain [29] (Fig. 2A&B). As described in the “Materials and Methods”, multiple tests of a triple labeling protocol using a rabbit anti-Ki67 antibody to replace the mouse anti-Ki67 antibody failed to reliably define Ki67 immunoreactivity in either the hypothalamus or the SVZ, although the mouse anti-nestin antibody applied simultaneously delineated the SVZ (Fig. 2A) as well as nestin-positive cells in the SDN-POA area of the hypothalamus (Fig. 2C–F). Nestin-immunoreactivity also labeled the microvasculature (Fig. 2). Similar to the SVZ, the nestin-immunoreactivity delineated a tube-like structure with a length of 360 µm starting at the rostral end of the 3^rd^ ventricle along the brain longitudinal axis in PND 21 and adult males (Figs. 3&4). Since the unaided human eye can resolve dimensions on the order of 100–300 µm, we propose to define the nestin-immunoreactivity delineated structure as “the macroscopic neural stem cell niche of the 3^rd^ ventricle.”

**Figure 5:**
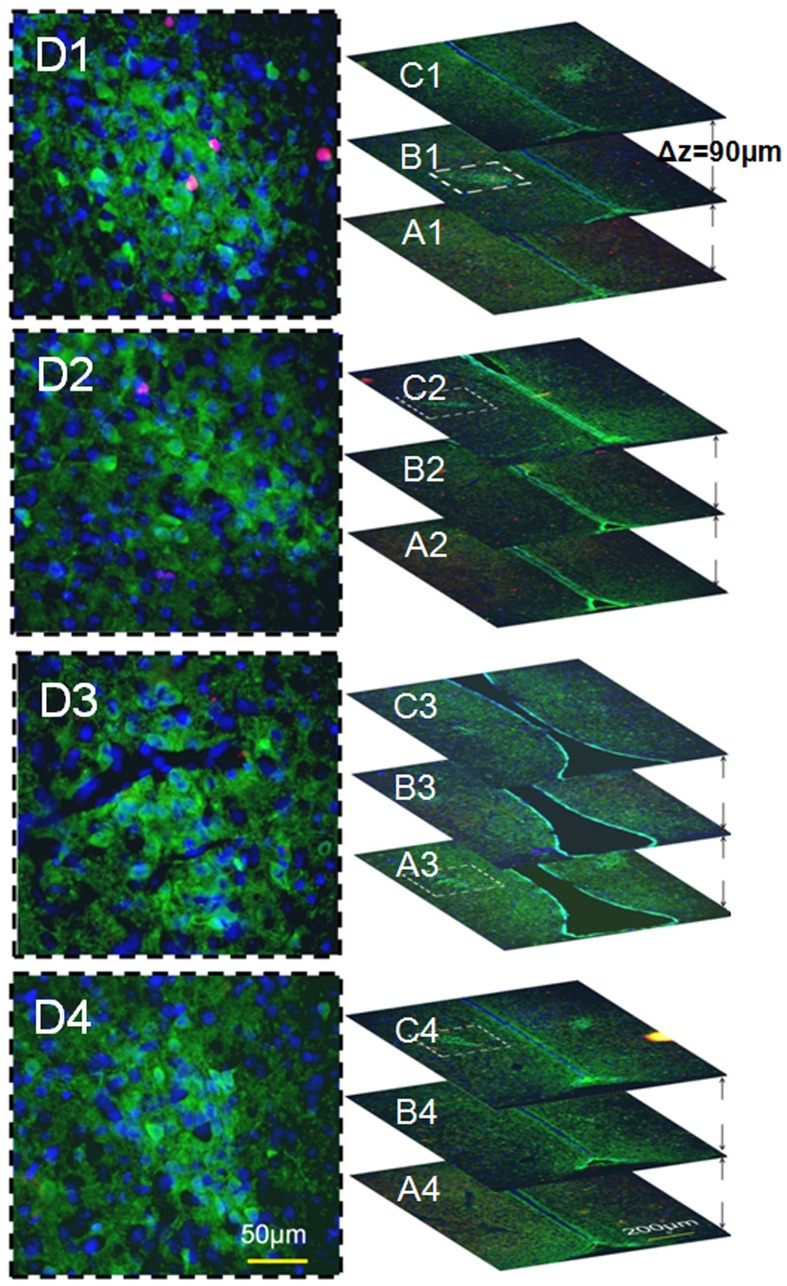
Representative images of the triple labeling of calbindin D28k (CB28), Ki67, and DAPI. Four sets of sequential images are displayed defining the SDN-POA (CB28-delineated, green fluorescence) and proliferative cells (Ki67-labeled, red fluorescence) in the hypothalamic preoptic area. The blue (DAPI) fluorescence delineates cell nuclei. The four series of images are from a PND 21 male (A1–D1), PND 21 female (A2–D2), PND 110 male (A3–D3), and PND 110 female (A4–D4). A to C images are aligned in a direction from rostral to caudal with 90 µm between adjacent slices along the longitudinal axis. Images displayed under higher magnification in D were acquired at the locations indicated by the white dotted area in their corresponding series.

**Figure 6:**
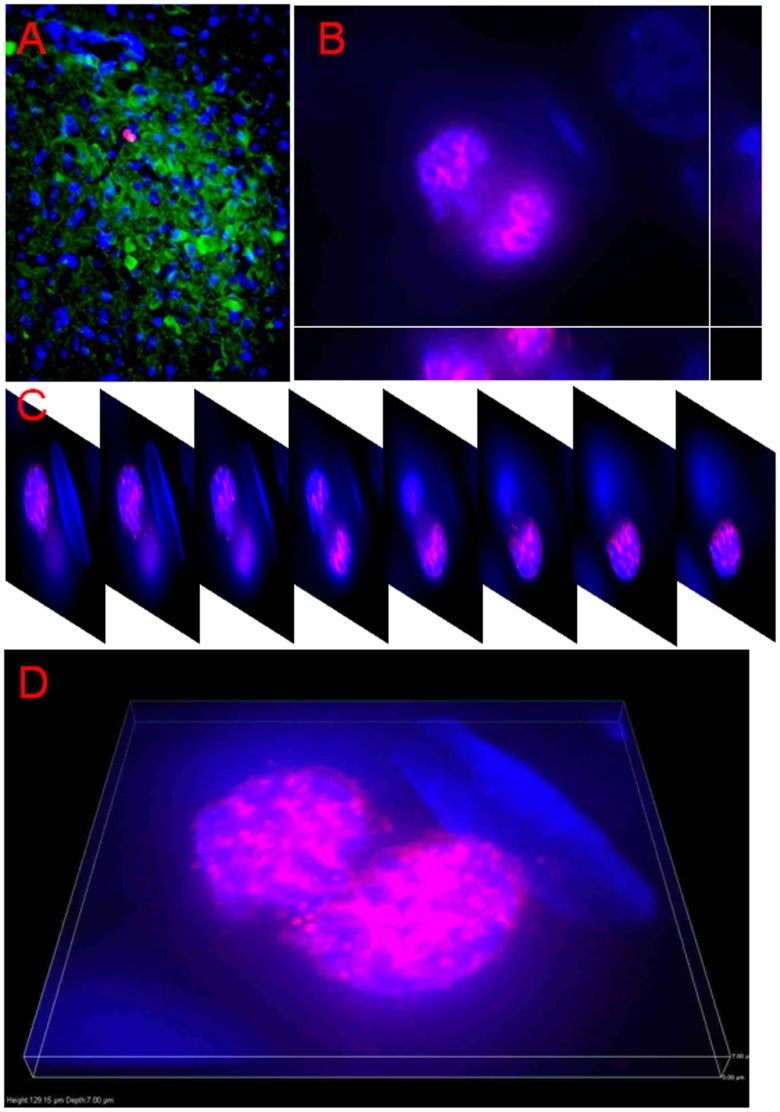
Representative images showing the morphology of dividing cells. A: This relatively low power image illustrates the CB28-immunoreactivity (green fluorescence) delineation of a unilateral adult male SDN-POA within which the red fluorescence (Ki67-immunoreactivity) is located. B, C and D demonstrate the cell at the telophase of cell division defined in A: B shows a cut view; C displays images with a sequential order at 1 µm intervals along the Z axis; and D highlights the 3-dimensional view with the stacked images.

**Figure 7:**
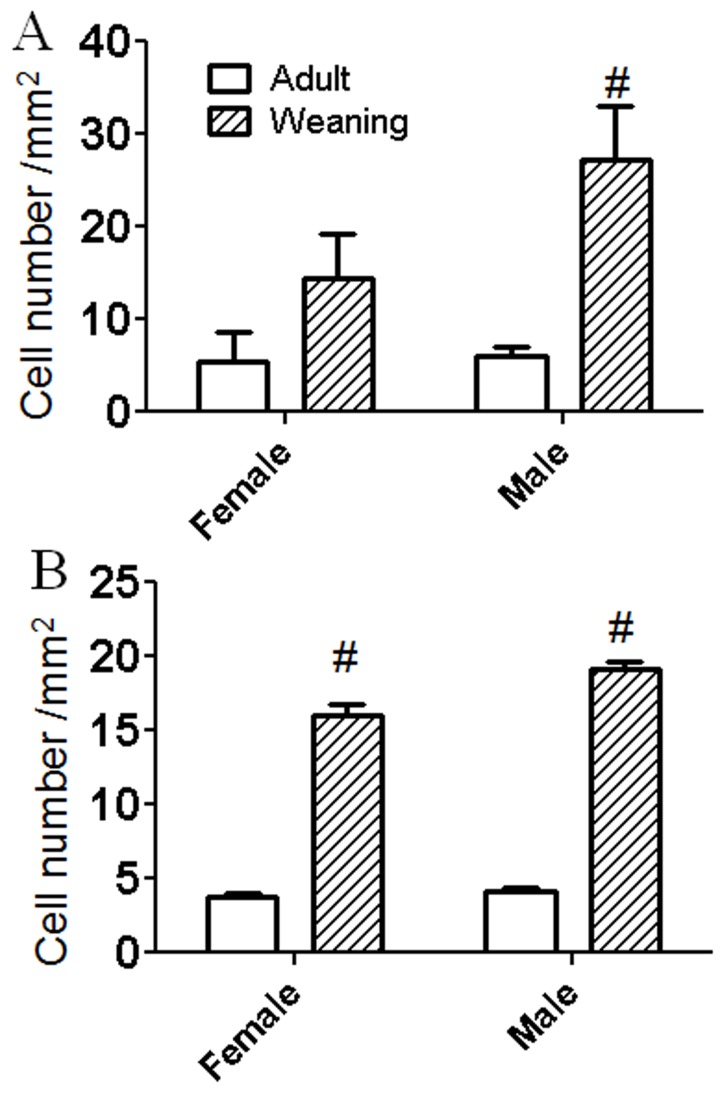
Ki67-positive cell counts in the SDN-POA (top) and hypothalamus (bottom). In the SDN-POA area, the number of Ki67-positive cells was 3.4 times higher at weaning than at adulthood in male rats (p<0.01), whereas the number in females was not statistically significantly different between the weaning group and the adult group (p>0.05). In the hypothalamus, the Ki67-positive cell number was 3.5 and 3.3 times higher respectively, in PND 21 male and female rats (p<0.001 for both) compared to same-sex PND 110 rats. #suggests age difference.

Mouse-monoclonal antibody against Ki67 worked well using either a double-labeling protocol to delineate the SVZ (Ki67-DAPI, Fig. 2B) or a triple labeling protocol to locate Ki67-positive cells within the SDN-POA and surrounding hypothalamic area (Ki67-CB28-DAPI, Fig. 5&6) as well as in the SVZ (data not shown). The Ki67-immunoreactivity was not observed in the mature neurons delineated by CB28-immunoreactivity (Fig. 5), which was verified by 3-dimensional views (data not shown). Ki67 immunoreactivity was generally confined to the nucleus, an organelle structure, which was delineated by the DAPI fluorescence-labeling. The double-labeling method (Ki67-DAPI) illustrated various dividing cell morphologies, including doublets and cell division phases from anaphase to telophase (Fig. 6), in weanlings and adults. In the SDN-POA area, the number of Ki67-positive cells was 3.4 times higher at weaning than at adulthood in male rats (p<0.01), whereas the number in females was not statistically different between the weanling group and the adult group (Fig. 7A). Neither the main effect of sex nor the interaction of sex and age was significant, possibly due to the limited animal number/group and high variability in the measure. In the hypothalamus (Fig. 7B), Ki67-positive cell numbers were 3.5 and 3.3 times higher, respectively, in PND 21 males and females relative to adults (p<0.001 for both). There was a significant interaction of sex and age (p<0.05), suggesting that sex and age might affect the Ki67-positive cell numbers in the hypothalamus.

## Discussion

### Formation of the SDN-POA

The neurons of the SDN-POA originate from the subependymal lining of the 3rd ventricle and migrate upward and laterally to arrive at and form the SDN-POA [30]. Using the thymidine analog bromedeoxyuridine (BrdU) to label cell proliferation, neurogenesis in the SDN-POA appears to occur on embryonic days 17–18 and this does not appear to be sexually dimorphic [13], [31]. Still, sexual dimorphism of the Nissl-stained SDN-POA was reported to be detectable as early as PND 1 [32]; however, a more recent study indicated no sex differences in Nissl-stained SDN-POA at PND 4 [31]. By PND 8, males have a significantly larger SDN-POA whether this is measured using Nissl-staining or CB28 labeling [11], [31]. Essentially, sexual dimorphism of the SDN-POA results from differences in postnatal growth: the male SDN-POA expands continuously at least through young adulthood whereas the size of the female SDN-POA remains relatively stable [11], [13], [32]. As demonstrated in the present study, the volume of the CB28 immunoreactivity-delineated SDN-POA increased approximately 43% from PND 21 to adulthood in males. Although the age-related increase was somewhat larger in females (≈58%), this did not reach statistical significance. To our knowledge, this is the first report of age-related SDN-POA volume differences as delineated using CB28 immunoreactivity. However, volume changes can occur over relatively short periods even in adults as demonstrated using the conventional Nissl staining method. For example, male SDN-POA volume has been shown to increase between PND 60 and 88 and this increase was unaffected by castration at PND 60 although castration did reduce SDN-POA soma area [33]. As shown in Fig. 5 & 7A, proliferation activity indicated by Ki67-positive cell counts in the SDN-POA appears relatively similar between males and females at weaning. If those Ki67-positive cells then differentiate into CB28-positive neurons in the SDN-POA of male rats (although this is uncertain), then the destination and/or fate of Ki67-positive cells in female SDN-POA may be different (see below concerning apoptosis). Interestingly, a recent report indicates that although adult male mice have more CB28-positive SDN-POA cells and surrounding areas than females, developmental cell death does not account for that sexual dimorphism [12]. As demonstrated in Fig. 7B, there is a sex difference in Ki67-positive cell counts of the hypothalamus, indicating the potential role of postweaning proliferation/neural stem cell activity in distinguishing the male and female SDN-POA.

### Developmental Mechanisms that Shape the SDN-POA

#### Migration

Cell migration is involved in shaping the SDN-POA during the perinatal period. The principal component of the SDN-POA (i.e., neurons) originates from the subependymal lining of the 3rd ventricle [30]. Relative to females, male mice have a faster rate of *in vitro* cell migration in a medial–lateral orientation in the preoptic area/anterior hypothalamus [34]. A study demonstrated that neural progenitor cells exist in the ependymal layer of the 3rd cerebral ventricle of adult rats and that they migrate and differentiate into functioning neurons in the hypothalamus [35]. Accordingly, those cells positive for Ki67 and/or nestin in the present study (Figs. 2&5) may have originated in the 3rd ventricle stem cell niche (Figs. 3&4) and migrated into the SDN-POA. Such cell migration may continue to affect the anatomical structure of the SDN-POA even during adulthood.

#### Apoptosis

Increased apoptosis in females was thought to be involved in shaping the sexual dimorphism of the SDN-POA since during PNDs 7–10, female rats display higher apoptotic cell counts than males [36], [37]. Further, testosterone significantly inhibits this apoptotic cell death during PNDs 6–10 and its effect is specific to the SDN-POA. No sex difference in apoptosis was detectable in the control region, the lateral preoptic area, and testosterone did not affect apoptosis in that area [36]. Sex differences in apoptotic cell number during development were inversely correlated with the number of adult cells in the SDN-POA [38]. Nevertheless, the sex difference in SDN-POA apoptosis exists only between PNDs 7–10 [36], whereas the SDN-POA continues to increase in male rats after this time [11], [33] (see Fig. 1A2). As suggested, sex differences in apoptosis may contribute little to the eventual development of the SDN-POA [12], [39].

#### Neural stem cell activity

The present study provides evidence that Ki67- and nestin-positive stem cell activity, such as proliferation/mitosis, exists in the postweaning male and female SDN-POA. In addition, we defined a gross-anatomic, neural stem cell niche of the 3^rd^ ventricle that exists in both young and adult animals (Fig. 3 & Fig. 4). These results add substantial information to the body of knowledge about SDN-POA development in the rodent. Whether the Ki67-positive cells are capable of differentiation into CB28-positive neurons remains unclear. However, because some Ki67-positive cells displayed the morphology of dividing cells, those cells would be capable of generating new cells to expand and enlarge the SDN-POA. Thus, postweaning stem cell activity may be one of the driving forces which shape the SDN-POA.

Stem cell activity and/or neurogenesis have been identified in the hypothalamus of rodents and humans during development and in adulthood [17], [40]–[43]. “Third ventricle germinal zones” [17] or the “periventricular zone of the 3^rd^ ventricle” [43] are proposed to be one of the sources where stem cell activity and/or neurogenesis originate. Cells in the SDN-POA of ferrets have been shown to migrate from proliferative zones lining the lateral as well as the third ventricle using a combined analysis defining the orientation of radial glial processes paired with quantitative, computer-assisted image analysis of BrdU-immunoreactivity [44]. Those reports define the stem cell niche, the third ventricle germinal zones [17], or the third ventricle periventricular zone [43], at the microscopic and/or ultramicroscopic level. On the other hand, the 3^rd^ ventricle extends in rats from Bregma 0.00 mm to Bregma −4.80 mm along the longitudinal axis of the brain [45]; the location at which stem cell activity can be reproducibly defined along the 4.80 mm long, tube-like structure of the 3^rd^ ventricle remains unclear. Using immunoreactivity to nestin, a protein marker for neural stem cells, the present study delineates for the first time at the macroscopic level, a three dimensional area of the 3^rd^ ventricle stem cell niche. This area is limited by the rostral end of the 3^rd^ ventricle and occupies less than 7.5% of its length along the brain’s longitudinal axis in rats. Because the concept of “third ventricle germinal zones” has been proposed for mice but no anatomical coordinates have been provided for them [17], we are hesitant to make any comparisons between our results and the study conducted by Dahiya et al. [17]. On the other hand, we, in the present study and others (Pérez-Martín, et al. [43]) have provided coordinates for rats: Bregma 0.00 to −0.36 mm (location of the macroscopic stem cell niche of the 3^rd^ ventricle) and Bregma −1.30 mm to −3.60, respectively, indicating two different locations. One explanation for this discrepancy is that Perez-Martín et al. [43] examined rats after intracerebroventricular treatment with insulin-like growth factor I and did not test for nestin-immunoreactivity, while the present report studied physiologically normal animals using nestin-immunoreactivity in areas down to Bregma −0.810 mm along the longitudinal axis of the 3^rd^ ventricle: no nestin-immunoreactivity was found longitudinally between Bregma −0.450 mm to −0.810 mm (Fig. 2 vs. Fig. 3). Further studies are required to explore the location of nestin-immunoreactivity in the caudal section of the 3^rd^ ventricle.

Neurogenesis has been proposed as a mechanism for actively maintaining sexual dimorphisms in cell number rather than passively sustaining them throughout life once the sexually dimorphic structures–including the SDN-POA–are established perinatally. Pubertal hormones contribute to the postnatal preservation of sexual dimorphisms via modulation of new cells that are added to sexually dimorphic brain regions [40]. As mentioned above, neural stem cell activity has been identified in the hypothalamus [17], [40]–[44], an area in which the SDN-POA is included. Nevertheless, to our knowledge, it remains unclear whether differences in stem cell activity between males and females in the perinatal period account for establishing the sexual dimorphism of the SDN-POA. It is worthy of mention that Ahmed et al. [40] determined neural stem cell activity in the SDN-POA by examining adjacent brain slices, one assessed histologically using Nissl stain to define the outline/volume of the sexually dimorphic structures and the other assessed using BrdU labeling to verify stem cell activity. However, the reported size of the SDN-POA using the conventional Nissl staining methods (thionin or cresyl violet) has varied by up to 7 and ∼30-fold for male and female rats, respectively [2]. Presumably, the lack of a clear-cut SDN-POA boundary identifiable in tissue stained using conventional histological methods accounts for such variation and, thus, the immunohistochemical identification of the SDN-POA using CB28 appears to be more specific and accurate [5]. The present study advances our understanding of the role of neural stem cell activity in the development of sexual dimorphism of the SDN-POA in three ways. First, neural stem cell activity/proliferation may be responsible for establishment of the sexual dimorphism of the SDN-POA since the Ki67-positive cell count in the SDN-POA in males is significantly higher at weaning than at adulthood whereas in females, the number at weaning is not statistically significantly greater compared with that at adulthood. In addition, a significant interaction of sex and age suggests that sex and age affect the number of Ki67-positive cells in the hypothalamus (Fig. 7B), indirectly suggesting that different numbers of Ki67-positive cells may be available for migration into the SDN-POA from surrounding areas of the hypothalamus: this could play a role in establishing the sexual dimorphism of the SDN-POA. The age at weaning age (PND21) is close to the initial time (PND8) when sexual dimorphism of the SDN-POA is discernible using the CB28 immunoreactivity approach in rats; in addition, the CB28 immunoreactivity-delineated SDN-POA continues to expand in male rats beyond weaning [11]. Secondly, stem cell activity/proliferation is substantial in the SDN-POA as evidenced by the fact that cells positive for Ki67, a marker of cellular proliferation, were observed within the SDN-POA as delineated by CB28 immunoreactivity (Fig. 5). Finally, neural stem cells retain their capacity to proliferate as evidenced by their ability to divide (Fig. 6), although it remains unknown whether they migrate into the targeted structures (here, the SDN-POA) after being activated in the stem cell niche.

### Summary: Addressing Significance of the Present Findings

Results of the present study indicate that neural stem cell activity is a possible mechanism by which postnatal sexual dimorphism of the SDN-POA may be established and which enables postweaning SDN-POA development. Specifically, neural stem cell activity–such as proliferation as measured by cell count, the presence of cell-division morphology and labeling with the proliferative/mitotic marker Ki67–may contribute to the noted sex differences. Differentiating and migrating capabilities, that remain to be studied in future efforts, may also further define the eventual shape and/or size of the SDN-POA. Given that the male SDN-POA volume continues to increase postweaning and that stem cell activity/proliferation was especially robust in weanlings relative to adults, the postweaning period may be critical for the development of the SDN-POA. Further, this postweaning stem cell activity may be sensitive to sex hormones and those exogenous compounds which mimic sex hormones. Additionally, definition of the macroscopic, three dimensional stem cell niche of the 3^rd^ ventricle is novel, providing increased fundamental knowledge concerning normal development of the surrounding structures and the potential for pathology (e.g., tumor development in the 3^rd^ ventricle) as well.

## Disclaimer

This document has been reviewed in accordance with United States Food and Drug Administration (FDA) policy and approved for publication. Approval does not signify that the contents necessarily reflect the position or opinions of the FDA nor does mention of trade names or commercial products constitute endorsement or recommendation for use. The findings and conclusions in this report are those of the author(s) and do not necessarily represent the views of the FDA.
